# Melatonin Suppresses Ferroptosis Induced by High Glucose via Activation of the Nrf2/HO-1 Signaling Pathway in Type 2 Diabetic Osteoporosis

**DOI:** 10.1155/2020/9067610

**Published:** 2020-12-04

**Authors:** Hongdong Ma, Xindong Wang, Weilin Zhang, Haitian Li, Wei Zhao, Jun Sun, Maowei Yang

**Affiliations:** ^1^Department of Orthopedics, The First Hospital of China Medical University, Shenyang, Liaoning, China; ^2^Department of Orthopedics, The Fourth Hospital of China Medical University, Shenyang, Liaoning, China; ^3^Department of Orthopedics, The Third Hospital of Jinzhou Medical University, Jinzhou, Liaoning, China

## Abstract

Ferroptosis is recently identified, an iron- and reactive oxygen species- (ROS-) dependent form of regulated cell death. This study was designed to determine the existence of ferroptosis in the pathogenesis of type 2 diabetic osteoporosis and confirm that melatonin can inhibit the ferroptosis of osteoblasts through activating Nrf2/HO-1 signaling pathway to improve bone microstructure *in vivo* and *in vitro*. We treated MC3T3-E1 cells with different concentrations of melatonin (1, 10, or 100 *μ*M) and exposed them to high glucose (25.5 mM) for 48 h *in vitro*. Our data showed that high glucose can induce osteoblast cytotoxicity and the accumulation of lipid peroxide, the mitochondria of osteoblast show the same morphology changes as the erastin treatment group, and the expression of ferroptosis-related proteins glutathione peroxidase 4 (GPX4) and cystine-glutamate antiporter (SLC7A11) is downregulated, but these effects were reversed by ferroptosis inhibitor ferrastatin-1 and iron chelator deferoxamine (DFO). Furthermore, western blot and real-time polymerase chain reaction were used to detect the expression levels of nuclear factor erythroid 2-related factor 2 (Nrf2) and heme oxygenase-1 (HO-1); osteogenic capacity was evaluated by alizarin red S staining and the expression of osteoprotegerin, osteocalcin, and alkaline phosphatase; the results showed that the expression levels of these proteins in osteoblasts with 1, 10, or 100 *μ*M melatonins were significantly higher than the high glucose group, but after using Nrf2-SiRNA interference, the therapeutic effect of melatonin was significantly inhibited. We also performed *in vivo* experiments in a diabetic rat model treated with two concentrations of melatonin (10, 50 mg/kg). Dynamic bone histomorphometry and micro-CT were used to observe the rat bone microstructure, and the expression of GPX4 and Nrf2 was determined by immunohistochemistry. Here, we first report that high glucose induces ferroptosis via increased ROS/lipid peroxidation/glutathione depletion in type 2 diabetic osteoporosis. More importantly, melatonin significantly reduced the level of ferroptosis and improved the osteogenic capacity of MC3T3-E1 through activating the Nrf2/HO-1 pathway *in vivo* and *in vitro*.

## 1. Introduction

Type 2 diabetes mellitus (T2DM) is a slowly progressing disease with substantial comorbidities, including neuropathy, nephropathy, and retinopathy. Epidemiological studies showed that the incidence of osteoporotic fractures is increased in patients with T2DM compared with the healthy population [1, 2], and this risk is further increased in patients treated with insulin [3]. However, the increased incidence of osteoporotic fractures cannot be explained by a decrease in bone mineral density (BMD), and in contrast to type 1 diabetes, the role of BMD in patients with T2DM is still unclear [4, 5]. The mechanism of T2DM-related osteoporosis (T2DOP) therefore remains to be clarified, to guide further clinical treatment.

Iron overload has been reported to lead to bone loss in mice, and this is closely related to osteoporosis [6–8]. Ferroptosis has recently been identified as an iron- and reactive oxygen species- (ROS-) dependent form of regulated cell death [9], reportedly involved in a variety of diseases, such as cancer, kidney degeneration, intracerebral hemorrhage, and traumatic brain injury [10–13]. This form of iron-dependent cell death differs from apoptosis, autophagy, necrosis, and other modes of cell death in terms of its biochemistry and morphology. Ferroptosis can be caused by specific inducers, such as elastin and RSL3. It is characterized by the accumulation of intracellular iron and lipid ROS, and its morphology is characterized by mitochondrial shrinkage [9, 14, 15]. Numerous studies have confirmed an important role for oxidative stress in the pathogenesis of diabetic osteoporosis [16–18]. We previously determined that iron overload is caused by overexpression of the divalent metal transporter 1 (DMT1) in osteoblasts and play an important role in the pathogenesis of T2DOP [19, 20]. We speculated that ferroptosis may occur during the onset of T2DOP and conducted the current study to determine the existence and mechanism of ferroptosis.

Melatonin (N-acetyl-5-methoxytryptamine) has many effects on the human body, including regulation of the sleep-wake rhythm, circadian cycle, immune defense, cardiovascular function, and bone metabolism [21–24]. In addition, numerous studies have demonstrated that melatonin is an effective endogenous antioxidant [25], which also indirectly stimulates certain antioxidant enzymes, such as superoxide dismutase (SOD) and glutathione peroxidase (GPx) [26]. Moreover, melatonin effectively prevents osteoporosis in ovariectomized rats and increases the volume of newly formed cortical femoral bone, promotes bone formation, and prevents bone loss in perimenopausal women [27, 28]. Our study confirmed the relationship between melatonin and autophagic cell death in T2DOP [19], and ferroptosis is a cell death mode closely related to ROS and iron homeostasis. Therefore, we speculated that melatonin and ferroptosis are correlated, but the specific mechanism remains to be further explored.

The nuclear factor erythroid 2-related factor 2 (Nrf2) signaling pathway is directly downstream of ROS and regulates the transcription of an antioxidant responsive element- (ARE-) dependent genes to balance oxidative mediators and maintain cellular redox homeostasis [29]. Recent research revealed that melatonin reduced kidney damage caused by diabetes and exerted neuroprotective effects by activating the Nrf2/heme oxygenase-1 (HO-1) pathway and increasing levels of the antioxidant enzymes HO-1 and NAD(P)H dehydrogenase [quinone] 1 (NQO1) [30, 31]. Nrf2 has also been reported to protect against ferroptosis induced by erastin or RSL3 in cancer cells [32, 33]. However, further studies are required to identify the potential mechanism of the Nrf2/HO-1 pathway and ferroptosis in the improvement of bone microstructure by melatonin.

We previously discovered that melatonin reduced autophagy in osteoblasts and delayed diabetes-induced osteoporosis by inhibiting the extracellular signal-regulated kinase signaling pathway [34]. Increasing evidence has also indicated that melatonin can regulate cell death pathways, including apoptosis, autophagy, and necrosis [35–37]. However, the ability of melatonin to protect osteoblasts against ferroptosis-induced damage in T2DOP, as well as the underlying mechanisms, remains unknown. The present study aimed to evaluate the effects of melatonin in *in vivo* and *in vitro* models of T2DOP and to determine if melatonin protected osteoblasts by inhibiting ferroptosis through relieving oxidative stress via the Nrf2/HO-1 pathway, as well as identifying potential therapeutic targets for clinical treatment.

### 1.1. Cell Culture

The osteoblastic cell line MC3T3-E1 was obtained from the American Type Culture Collection. Cells were cultured in *α*-minimum essential medium (*α*-MEM) (Hyclone, UT, USA) containing 10% fetal bovine serum (Hyclone) in a humidified 5% CO_2_ atmosphere at 37°C. MC3T3-E1 cells were cultured in T2DM-mimic conditions in osteogenic *α*-MEM with 25.5 mM glucose (Sigma-Aldrich, MO, USA) and 1 mM free fatty acids (palmitic acid and oleic acid at a ratio of 1 : 2). MC3T3-E1 cells were incubated in regular culture medium containing different concentrations of melatonin (0, 1, 10, or 100 *μ*M) for 48 h after exposure to high glucose (HG, 25.5 mM) for 24 h. To investigate the existence and potential mechanism of ferroptosis in osteoblasts, the ferroptosis inhibitor ferrostatin-1 (Fer-1) (5 *μ*M), autophagy inhibitor 3-methyladenine (3-MA) (5 mM), apoptosis inhibitor Z-VAD-FMK (10 *μ*M), and necrosis inhibitor necrostain-1 (Nec-1) (50 *μ*M) were added to the cell cultures.

### 1.2. Reagents and Antibodies

Melatonin, bovine serum albumin, deferoxamine (DFO), Fer-1, 3-MA, Z-VAD-FMK, Nec-1, and luzindole (a melatonin receptor antagonist) were purchased from Sigma-Aldrich (St. Louis, MO, USA). *NRF2* small interfering RNA (siRNA) was purchased from GeneChem (China). The primary antibodies used were as follows: anti-osteoprotegerin (OPG, ab73400), anti-GPx4 (ab125066), anti-SLC7A11 (ab37185), anti-Nrf2 (ab62352), anti-NQO1 (ab80588), anti-HO1 (ab13248), and anti-osteocalcin (OCN, ab13420) (all from Abcam, Cambridge, UK). All the secondary antibodies and Anti-glyceraldehyde-3-phosphate dehydrogenase (GAPDH, TA309157) were purchased from ZsBio.

### 1.3. Cell Viability Analysis

Take logarithmic growth phase cells, digest and make a single cell suspension and count, inoculate MC3T3-E1 cells in a 96-well plate according to the cell density of 5000/well, and add PBS to the edge wells for liquid sealing. When the fusion rate of the cells rises to 70-90%, the original medium is discarded and changed to a serum-free medium, and the cells are starved for 24 h to promote cell synchronization. After adding 10*μ*LCCK8 reagent to each well and reacting in a 37°C incubator for 4 h, the microplate reader (Thermo, MA, USA) was used to detect the OD value of cells in each well at 450 nm. Relative cell activity = (treatment group OD value-blank group OD value)/(control group OD value-blank group OD value) and expressed as a percentage. Each experiment was repeated three times.

### 1.4. siRNA Transfection

*NRF2* gene expression was knocked down by transfecting 1 × 10^6^ MC3T3-E1 cells with siRNA-NRF2 or siRNA control (GenePharma Co. Ltd., Shanghai, China). Transient siRNA transfection was performed using Lipofectamine 3000 (Invitrogen, USA), according to the instructions of the manufacturer. After 24 h of transfection, MC3T3-E1 were treated with HG and different concentrations of melatonin. The transfection efficiency was confirmed by western blotting.

### 1.5. Determination of ROS Levels

Cells were seeded at 2 × 10^5^ cells in 6-well plates. Added 2 mL medium with the different drugs and incubated for 48 h, and replaced the culture medium with serum-free medium, which is contained 10 *μ*mol/L 2′,7′-dichlorodihydrofluorescein diacetate (Sigma-Aldrich, St. Louis, MO, USA) and placed in the dark for 30 min. Add 5 *μ*L of the preconfigured 7-aminoactinomycin D (KeyGEN Bio-TECH, Nanjing, China) solution to each tube and incubate at room temperature in the dark for about 5 min to remove some dead cell interference. Finally, it was detected on the flow cytometer, using an emission wavelength of 525 nm and an excitation wavelength of 488 nm. The average fluorescence intensity of the cells in each group represented the level of ROS in the cells.

### 1.6. Determination of Reduced Glutathione (GSH) Levels

MC3T3-E1 cells were seeded at 2 × 10^5^ cells in 6-well plates. After 48 hours of treatment with different methods, the cells were washed with phosphate-buffered saline (PBS) and centrifuged twice to collect the cells. Three times the volume of the cell particles were added to the protein removal reagent solution and then perform two quick freeze-thaw cycles on the sample using liquid nitrogen and a 37°C water bath, respectively, followed by 4°C for 5 min and centrifuged at 10,000 × *g* for 10 min. Take the supernatant and determine the glutathione content according to the method provided by the GSH and GSSG Assay Kit (Product No. S0053, Beyotime). The absorbance of each well was measured at a wavelength of 410 nm on a microplate reader. Draw a standard curve according to the absorbance of the standard product and finally calculate the GSH concentration of each group of samples.

### 1.7. Malondialdehyde (MDA) Measurement

Carefully extract each group of cellular proteins and add 0.1 mL sample and 0.2 mL MDA detection working solution to each well. After mixing, heat in boiling water for 15 minutes. After cooling, 200 *μ*l of supernatant was added to a 96-well plate, and then, the absorbance was measured at 532 nm using a microplate reader to calculate the MDA level.

### 1.8. Real-Time Reverse Transcription (RT)-Polymerase Chain Reaction (PCR)

Total RNA was isolated using MiniBEST Universal RNA Extraction Kit (Takara, Shiga, Japan), and reverse transcription was performed using Primescript RT Master Mix (Takara). Real-time PCR was performed on the LightCycler 480 real-time PCR system (Roche, Basel, Switzerland) using the SYBR premix ex taq II kit (Takara). The table lists the primers used to amplify GPx4 (*GPX4*), Nrf2 (*NRF2*), HO-1 (*HMOX1*), alkaline phosphatase (ALP, *ALPL*), OPG, OCN, and *β*-actin (*ACTB*). The amplification conditions are as follows: 95°C for 30 s, followed by 40 cycles of 95°C for 5 s, and 60°C for 20 s. Relative mRNA expression was quantified by comparing cycle threshold (Ct) values, and *β*-actin was used as an internal control. The experimental data were processed by the 2^-*ΔΔ*Ct^ method as follows: *ΔΔ*Ct = (Ct target-Ct internal control) experimental group-(Ct target-Ct internal control) control group [38]. Each experiment was repeated three times.

### 1.9. Western Blot Analysis

After treatment, first, carefully aspirate the treated cell supernatant from each group and add 2 mL of PBS for repeated washing; then, the cells were extracted with lysis buffer and place on ice to lyse for 30 min. The supernatant was centrifuged at 12,000 × *g* for 15 min at 4°C, and the supernatant containing total protein was harvested. Electrophoresis and transferred to polyvinylidene difluoride membranes according to different molecular weights of each protein at low temperature. After blocking, place it in the primary antibody incubation box at a dilution ratio of 1 : 1000 and overnight at 4 degrees Celsius. Then, carefully move the membranes into the secondary antibody incubation box, add goat anti-rabbit and anti-mouse secondary antibodies at a dilution ratio of 1 : 5000, and incubate for 1 hour at room temperature. Specific band was visualized using the EC3 Imaging System (UVP Inc. Upland, CA, USA), and the optical density of each band was measured using the ImageJ software (NIH, Bethesda, MD, USA). The relative content was calculated as the ratio between the protein of interest and GAPDH in the same sample and expressed graphically.

### 1.10. Transmission Electron Microscopy

MC3T3-E1 cells were resuspended and transferred to 6-well plates to continue culturing for 48 hours with different intervention methods, the collected cells were fixed with 0.25% Trypsin-EDTA (Gibco Life Technologies, Gaithersburg, MD, USA) for 4 hours, and 1% osmic acid (Sigma-Aldrich) ∗ 0.1 M phosphate buffer (PH7.4) was fixed at room temperature for 2 hours. Stain the specimen with lead rafter acid (Sigma-Aldrich) and 2% uranium acetate (Amresco, Solon, OH, USA) saturated aqueous solution in sequence. Then, we use an Ultracut S ultramicrotome (Leica Microsystems, Wetzlar, Germany) to prepare 50 nm thick slices. Put the slice under the transmission electron microscope (JEOL, Ltd., Tokyo, Japan) and observe the ultrastructure of the cell under the electron microscope.

### 1.11. Alizarin Red S Staining

MC3T3-E1 cells were resuspended, inoculated, and cultured at a density of 1 × 10^5^/well. After 48 h of different treatments, the cells were cultured in osteogenic induction medium for 2 weeks. After rinsing, fix with 10% paraformaldehyde for 10 minutes. Prepare 1% alizarin red solution (Sigma-Aldrich) and stain for 5 minutes. After rinsing again, observe with an inverted optical microscope using Image-Pro Plus 6.0 (Media Cybernetics, Bethesda, MD, USA) and count the number of calcium nodules in ten fields.

### 1.12. Ethics Statement

The institutional Ethics Review Board of the First Hospital of China Medical University approved the study. The use of animals in our experiments was consistent with ethical requirements. All activities associated with this research project were performed following the First Hospital of China Medical University Institutional Guidelines and Clinical Regulations.

### 1.13. Experimental Animals

Eight-week-old specific-pathogen-free Sprague Dawley rats weighing 220 ± 20 g were purchased from China Medical University, Department of Experimental Animals (Animal Certificate Number: SCXK (Liaoning) 2008-0005). A total of 60 rats were used to determine the targets of bone histomorphometry; 45 rats were used to establish a diabetic model, and remaining 15 rats were divided into a control group. The diabetic rats were divided into three groups (*n* = 15 each) treated with intraperitoneal injection of high-dose melatonin (50 mg/kg, HMT group), intraperitoneal injection of low-dose melatonin (10 mg/kg, LMT group), and a control T2DM group.

### 1.14. Models and Specimen Collection

First, SD rats were fed with a high-fat and high-sugar diet for 2 months, and at the same time, they could drink water for 12 hours/day. In order to better construct a type 2 diabetes model and improve the success rate of modeling, we chose a small dose of streptozotocin (Sigma-Aldrich, S0130) (30 mg/kg) multiple intraperitoneal injection methods for modeling. After 72 hours, the fasting blood glucose was >7.8 mmol/L and insulin sensitivity decreased, and the model was successfully established [39]. Blood glucose and body weight were measured and recorded during a fixed period of time (0, 4, 8, and 12 weeks). The rats were housed in a temperature-controlled room (22.6 ± 2°C) with a light/dark cycle of 12 hours. The weight of the rats was maintained between 220 g and 270 g, and the blood glucose was maintained at 5 mmol/L and 18 mmol/L. The rats were sacrificed by cervical dislocation 4 W, 8 W, and 12 W after making the model, and the tibia was taken into sterile fresh 4% phosphate-buffered formalin solution and then stored in the refrigerator at 4°C.

### 1.15. Calcein Double-Labeling

Calcein double-labeling of bones was performed by intraperitoneal injection of calcein (5 mg/kg) at 12 and 2 days before sacrifice. Double calcein green labeling was measured on the cortical bone near the proximal metaphysis using fluorescence microscopy (Olympus BX-60, Tokyo, Japan). The mineral apposition rate (MAR), mineralizing surface/bone surface (BS), and bone-formation rate (BFR) were measured according to standardized protocols using an Osteo-Measure histomorphometry system (Osteometrics, Decatur, GA, USA). All the parameters followed the histomorphometric nomenclature and definitions of the American Society of Bone Mineral Research.

### 1.16. Microcomputed Tomography (CT) Assessment

The rats were sacrificed by cervical dislocation under anesthesia at 4, 8, and 12 weeks. The right femurs were obtained, and the soft tissue was removed. Bone samples were then placed in a 10-mm diameter tube perpendicular to the scanning axis, and the following scan parameters were selected: 1024 × 1024 image matrix, 80 kV voltage, 80 *μ*A current, 2.96 s exposure time. A cancellous bone area (1.0 mm by 3.0 mm thick) was selected from the distal growth plate, and the lowest threshold of 190 image extractions was used to make a line of reconstruction. The images were recombined via micro-CT, and the following parameters were determined: BMD, trabecular bone volume per tissue volume (BV/TV), trabecular number (Tb.N), and trabecular thickness (Tb.Th).

### 1.17. Immunohistochemistry (IHC)

Tibia sections (10 *μ*m) were deparaffinized in xylene and rehydrated in a graded series of ethanols (100%, 95%, 80%, and 75%) for 5 min each, followed by antigen recovery. The sections were then incubated in 3% H_2_O_2_ at 37°C for 15 min, washed with PBS, incubated with goat serum for 30 min at 37°C, and then incubated with primary rabbit monoclonal anti-GPx4 (1 : 100; ab125066, Abcam, Cambridge, MA, USA) or anti-Nrf2 antibody (1 : 100; ab62352, Abcam) at 4°C overnight. The next day, the sections were incubated with secondary goat anti-rabbit antibody (ZsBio, Beijing, China) for 45 min at 37°C followed by an ABC working solution (ZsBio) for 25 min at 37°C and incubated with 3,3-diaminobenzidine (ZsBio). Ten visual fields were randomly chosen for each sample under a LEICA DM600B automatic microscope (Leica Microsystems, Heidelberg GmbH, Germany). GPx4 and Nrf2 expressions were quantified using Image-Pro Plus 6.0 (Media Cybernetics), and the mean density value (integrated optical density divided by the relevant area) was calculated for each visual field.

### 1.18. Statistical Analysis

Quantitative variables were expressed as the mean ± standard deviation. All data were analyzed using GraphPad Prism 6.02 (GraphPad Software Inc., San Diego, CA, USA). Differences between two groups were analyzed by Student's *t*-test, and multiple groups were compared by one-way analysis of variance (ANOVA). A *P* value < 0.05 was considered statistically significant.

## 2. Results

### 2.1. Ferroptosis Was Induced by HG in MC3T3 Cells

**Figure 1:**
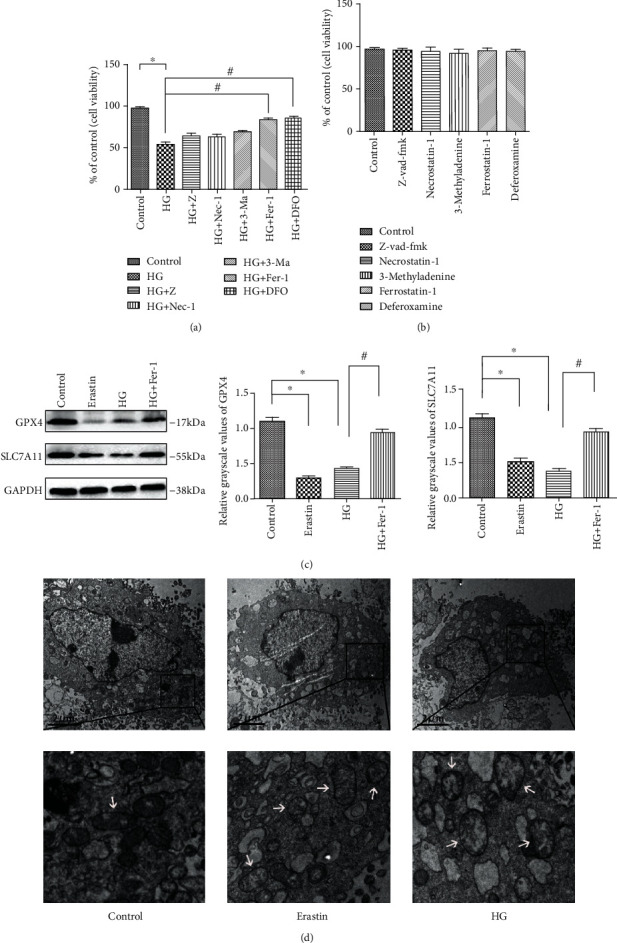
Ferroptosis is induced by high glucose in MC3T3 cells. (a) MC3T3 cells were cultured with Z-VAD-FMK (Z, 10 *μ*M), necrostain-1 (Nec-1, 50 *μ*M), 3-methyladenine (3-MA, 5 mM), ferrostatin-1 (Fer-1, 5 *μ*M), and deferoxamine (DFO, 10 *μ*M), respectively, followed by high glucose (HG, 25.5 mM) for 24 h. (b) MC3T3 cells were only cultured with Z-VAD-FMK, Nec-1, 3-MA, Fer-1, and DFO, Cell survival was tested by CCK-8 assay. (c) MC3T3 cells were treated with erastin (10 *μ*M) as a positive control. The ferroptosis-related proteins, GPx4 and SLC7A11, were determined by western blotting. (d) Transmission electron microscopy images of MC3T3 cells treated with HG (25.5 mM) and erastin (10 *μ*M), respectively, for 24 h. Arrows indicate mitochondria. Scale bars: 2 *μ*m; 0.5 *μ*m. Standard error represents three independent experiments (*n* = 3). ^∗^*P* < 0.05 vs. control, ^#^*P* < 0.05 vs. HG treatment.

Based on previous reports, we treated MC3T3 cells with 25.5 mM glucose (HG), consistent with the microenvironment of T2DM, and control cells were treated with 5.5 mM glucose [40]. HG significantly suppressed cell viability, as determined by CCK-8 assay (Figure 1(a)). To determine if the decrease in cell viability induced by HG was due to ferroptosis, we treated the cells with the caspase inhibitor Z-VAD-FMK and measured cell death. The caspase inhibitor failed to block HG-induced cell death (Figure 1(a)). Similarly, we treated HG MC3T3 cells with the autophagy inhibitor 3-MA and necrosis inhibitor Nec-1, which produced no significant improvement in cell survival (Figure 1(a)). However, treatment of the cells with Fer-1, which inhibits ferroptosis, blocked HG-induced cell death (Figure 1(a)). Ferroptosis is an iron-dependent type of cell death defined by lipid peroxidation. To identify the role of iron in HG-induced cell death, we treated MC3T3 cells with the iron chelator DFO, which resulted in an obvious increase in HG-induced cell death (Figure 1(a)). In contrast, the use of any of these reagents alone (Z-VAD-FMK, Fer-1, 3-MA, Nec-1, and DFO) had no effect on the viability of MC3T3 cells (Figure 1(a)).

**Figure 2:**
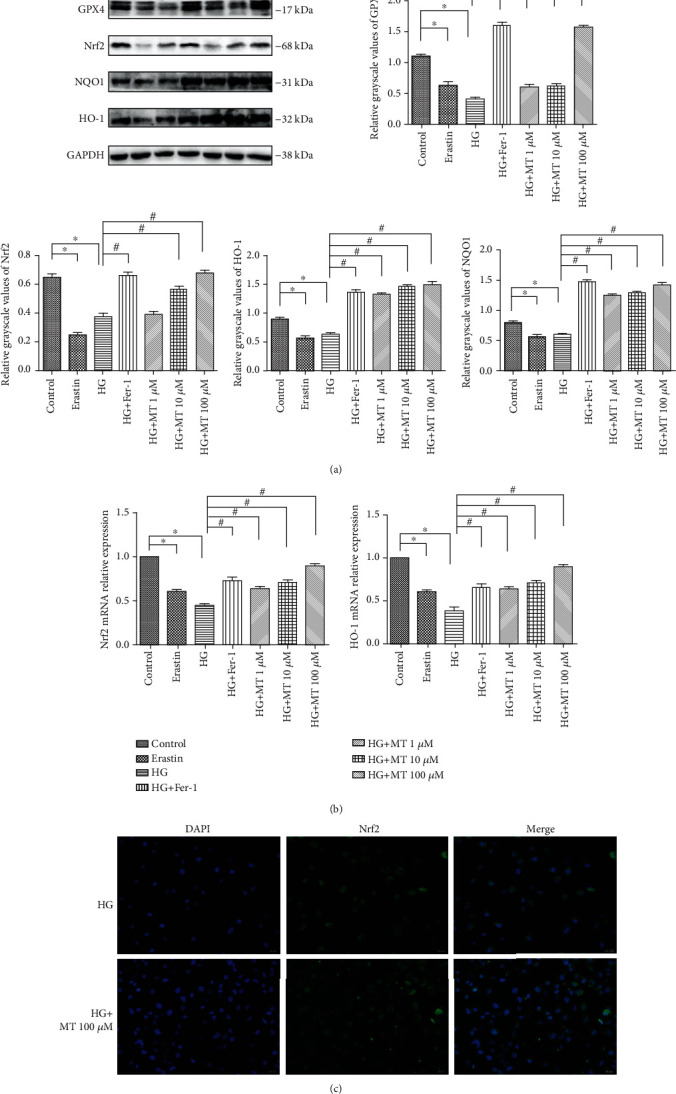
Melatonin suppresses ferroptosis via activation of Nrf2/HO-1 signaling pathway *in vitro*. (a) GPx4, Nrf2, NQO1, and HO-1 protein levels in MC3T3 cells treated with high glucose (HG, 25.5 mM), melatonin (1, 10, or 100 *μ*M), or both, determined by western blot. (b) Real-time reverse transcription-polymerase chain reaction analysis of NRF2 and HMOX1 mRNA expression levels in MC3T3 cells treated with HG (25.5 mM), melatonin (1, 10, or 100 *μ*M), or both for 48 h. (c) Immunofluorescence staining to detect the expression and location of NRF2 in MC3T3 cells treated with HG (25.5 mM) and melatonin (100 *μ*M). Standard error represents three independent experiments (*n* = 3). ^∗^*P* < 0.05 vs. control, ^#^*P* < 0.05 vs. HG treatment.

**Figure 3:**
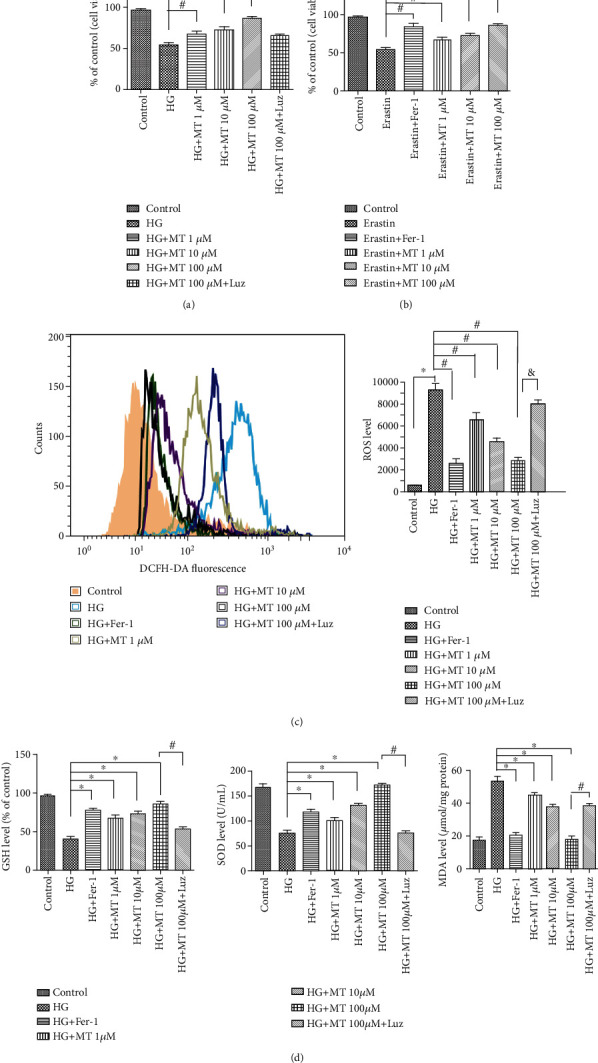
Melatonin improves MC3T3 cell viability by inhibiting lipid peroxidation. (a) MC3T3 cells were exposed to high glucose (HG, 25.5 mM) for 24 h before treatment with melatonin (1, 10, or 100 *μ*M) for 48 h. The melatonin receptor inhibitor luzindole (Luz, 5 *μ*M) was used in combination with the 100 *μ*M melatonin group. Cell survival was determined by CCK-8 assay. (b) MC3T3 cells were treated with erastin (10 *μ*M) instead of HG for 24 h before treatment with melatonin (1, 10, or 100 *μ*M) for 48 h, and cell survival was tested by CCK-8 assay. (c) ROS generation was demonstrated by flow cytometry with dihydroethidium (10 *μ*M) after MC3T3 cells were treated for 48 h. (d) Glutathione levels in MC3T3 cells after indicated treatments. Lipid peroxidation was determined by malondialdehyde and superoxide dismutase assays. Standard error represents three independent experiments (*n* = 3). ^∗^*P* < 0.05 vs. control, ^#^*P* < 0.05 vs. HG or erastin treatment, ^&^*P* < 0.05 vs. HG + 100 *μ*M melatonin treatment.

GPx4, as a marker of ferroptosis, was markedly decreased by HG treatment, consistent with the positive control (erastin) and the expression level of SLC7A11 (Figure 2(b)). Furthermore, the addition of Fer-1 to HG-treated osteoblasts significantly increased the expression levels of GPx4 and SLC7A11 compared with the HG-alone group (Figure 2(b)). Mitochondrial changes are considered to be a major feature of ferroptosis [9]. Accordingly, mitochondrial ultrastructural changes were observed in MC3T3 cells treated with HG and erastin, respectively, and mitochondria appeared generally smaller and less tubular, with a darker-stained membrane with distinct disrupted inner membrane folding (Figure 1(c)). Lipid ROS levels were measured using SOD and MDA kits, and ROS levels were measured by flow cytometry, indicating that lipid ROS levels were increased by HG treatment (Figures 3(c) and 3(d)). Overall, we concluded that ferroptosis was involved in the process of HG-induced cell death, suggesting that this novel mode of cell death could open up new treatment options for the clinical treatment of T2DM.

### 2.2. Melatonin Protected MC3T3 Cells against Ferroptosis via the Nrf2/HO-1 Signaling Pathway

We determined if MC3T3 cell viability was affected by HG-induced ferroptosis and rescued by melatonin supplementation. The HG-induced decrease in cell viability was reversed by 1, 10, and 100 *μ*M melatonin, as determined by CCK-8 assay (Figure 3(a)). To confirm that melatonin could increase cell viability, we showed that the addition of the melatonin receptor inhibitor luzindole to HG-treated cells reversed the therapeutic effect of melatonin (Figure 3(a)). Different concentrations of melatonin also increased cell viability after treatment with erastin (positive control) (Figure 3(b)).

**Figure 4:**
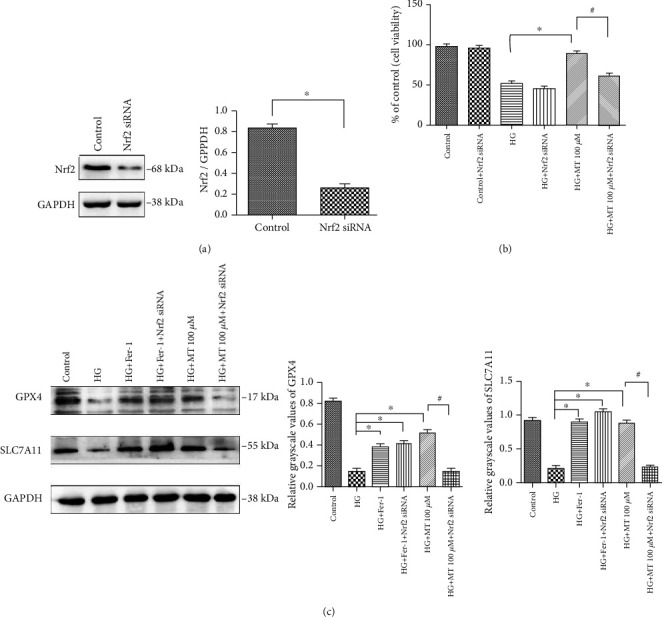
Inhibition of ferroptosis by melatonin can be regulated by NRF2-siRNA. (a) Nrf2 protein levels in MC3T3 cells demonstrated by western blotting. Histogram shows densitometry quantification of Nrf2 normalized to actin. (b) MC3T3 cells were exposed to high glucose (HG, 25.5 mM) and transfected with NRF2-siRNA for 24 h before treatment with melatonin (100 *μ*M). Cell survival was tested by CCK-8 assay. (c) GPx4 and SLC7A11 protein levels in MC3T3 cells after different treatments, demonstrated by western blotting. Standard error represents three independent experiments (*n* = 3). ^∗^*P* < 0.05 vs. HG treatment, ^#^*P* < 0.05 vs. HG + 100 *μ*M melatonin treatment.

Regarding lipid peroxidation, melatonin and Fer-1 significantly restored ROS levels and lipid peroxide generation, including GSH and MDA levels and SOD activity (Figure 3(d)). Flow cytometry also showed that melatonin significantly reduced ROS production, especially 100 *μ*M melatonin, but this improvement was inhibited by the melatonin receptor inhibitor luzindole (Figure 3(c)). Treatment of cells with melatonin and Fer-1 significantly increased the protein concentration of GPx4, indicating that melatonin and Fer-1 inhibited ferroptosis in MC3T3 cells (Figure 4(a)).

In addition to these functions, previous studies have demonstrated that melatonin was also an effective endogenous antioxidant [25, 41]. In the current study, melatonin was shown to scavenge ROS (Figure 3(c)). The Nrf2 signaling pathway is the direct downstream pathway of ROS and regulates the transcription of ARE-dependent genes to balance oxidative mediators and maintain cellular redox homeostasis. Nrf2, NQO1, and HO-1 expression were inhibited during HG- and erastin-induced ferroptosis, as shown by western blotting and RT-PCR. However, the expression levels of these proteins were significantly increased by treatment with different concentrations of melatonin and Fer-1 (Figures 2(a) and 2(b)), suggesting that melatonin inhibited ferroptosis by activating Nrf2/HO-1 pathways.

To further illustrate the role of the Nrf2 pathway in HG-induced ferroptosis, we knocked down *NRF2* expression using NRF2-siRNA. Western blotting confirmed a significant decrease in Nrf2 expression after transfection (Figure 4(a)). Melatonin increased the viability of NRF2-siRNA-transfected HG cells, as shown by CCK-8 assay (Figure 4(b)). Protein concentrations of GPx4 and SLC7a11 are important indicators of ferroptosis. We therefore detected these proteins in MC3T3 cells by western blotting and found no significant changes in their expression levels in the NRF2-siRNA group, suggesting that the ferroptosis inhibitor Fer-1 was not closely related to the Nrf2/HO-1 pathway (Figure 4(c)). However, 100 *μ*M melatonin significantly reduced the expression of GPx4 and SLC7a11 compared with the HG-alone group, suggesting that active expression of the Nrf2/HO-1 pathway was an important prerequisite for the inhibition of ferroptosis by melatonin (Figure 4(c)).

### 2.3. Melatonin Prevented HG-Induced Inhibition of Osteogenic Differentiation *In Vitro*

**Figure 5:**
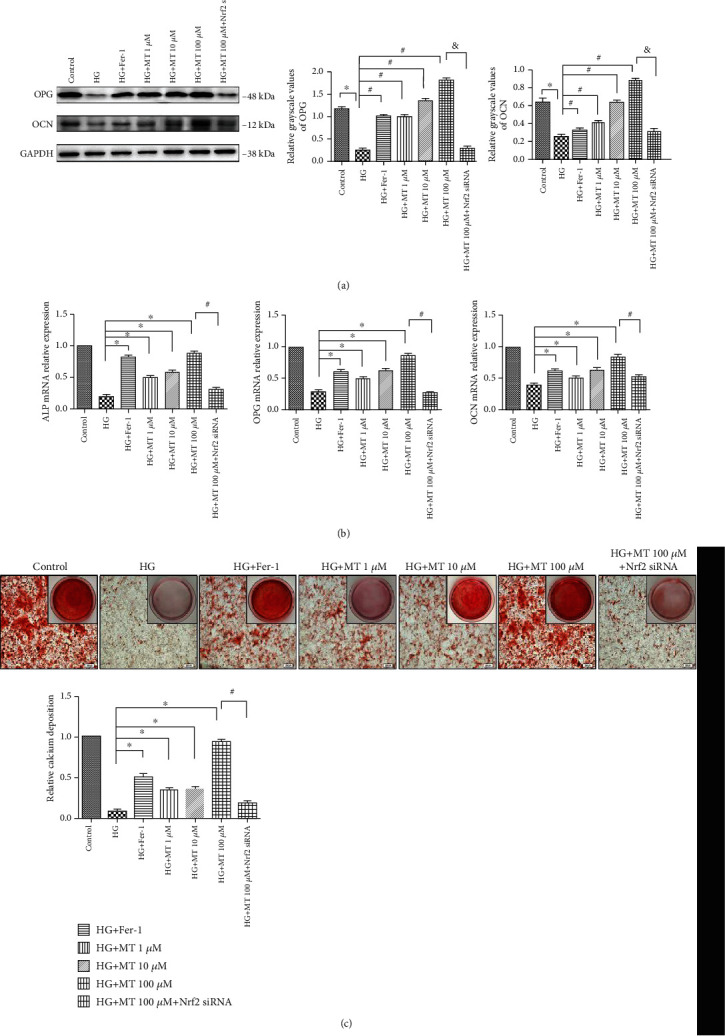
Melatonin improves osteogenic capability by suppressing ferroptosis in MC3T3 cells. (a) Osteoprotegerin (OPG) and osteocalcin (OCN) protein levels in MC3T3 cells detected by western blotting after high glucose (HG) and melatonin treatment (1, 10, or 100 *μ*M) for 48 h, then replace transfected cells in HG+ 100 *μ*M melatonin group. (b) Real-time reverse transcription-polymerase chain reaction analysis of ALP, OPG, and OCN mRNA expression in MC3T3 cells after indicated treatments. (c) Mineralized extracellular matrix in transfected MC3T3 cells after osteogenic differentiation for 14 days, shown by alizarin red S staining. Standard error represents three independent experiments (*n* = 3). ^∗^*P* < 0.05 vs. HG treatment, ^#^*P* < 0.05 vs. HG + 100 *μ*M melatonin treatment.

We assessed the osteoblast osteogenesis-associated proteins, OPG and OCN. OPG inhibits the function of osteoclasts, and reduced OPG expression can therefore serve as a biomarker of bone resorption. OCN promotes osteogenesis, is essential for bone mineralization, and is thus a biomarker of osteogenesis. OPG and OCN levels were upregulated in the HG plus melatonin 100 *μ*M group compared with the HG-alone group, while the opposite trend was observed in the NRF2-siRNA HG and 100 *μ*M melatonin group (Figure 5(a)). For further verification, we detected ALP, OCN, and OPG mRNA levels in MC3T3 cells by RT-PCR. Increasing the melatonin concentration significantly increased the osteoblastic ability of osteoblasts compared with the HG-alone group, but osteogenesis was significantly reduced in the NRF2-siRNA group (Figure 5(b)). We also assessed the effects of ferroptosis and melatonin on the ability of MC3T3 cells to differentiate into mature osteoblasts and form a mineralized extracellular matrix, using alizarin red S staining. After 14 days of culture, cells treated with HG and 100 *μ*M melatonin showed increased mineralized nodule formation compared with the HG-alone group, while the opposite result was observed in the HG plus NRF2-siRNA group (Figure 5(c)). These results suggested that, in the presence of HG, melatonin might be positively correlated with osteogenic ability via the Nrf2/HO-1 pathway.

### 2.4. Melatonin Augmented Bone Formation and Improved Bone Mass in T2DM Rats

**Figure 6:**
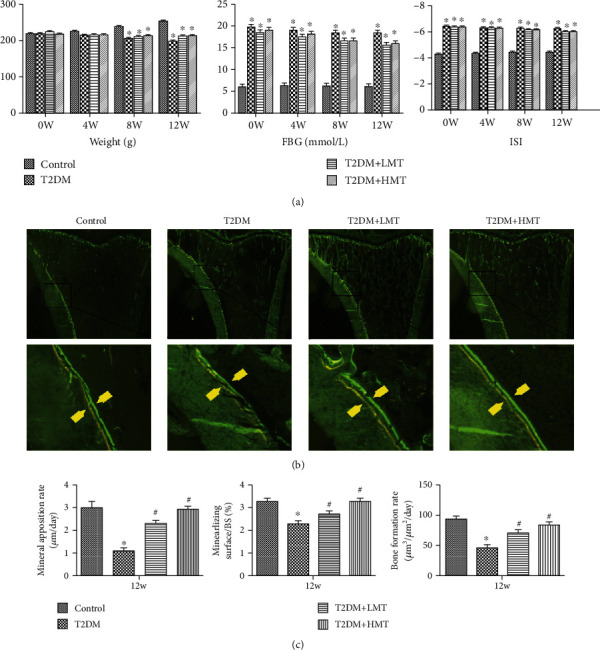
Melatonin increases mineral apposition and bone formation in type 2 diabetic osteoporosis. Forty-five model rats were divided into low-dose melatonin (LMT, *n* = 15, 10 mg/kg melatonin), high-dose melatonin (HMT, *n* = 15, 50 mg/kg melatonin), and control type 2 diabetes mellitus (T2DM, *n* = 15) groups. Fifteen nondiabetic rats were included as a control group. (a) Evaluation of T2DM model. Body weights were significantly lower in the model rats at 8 and 12 weeks, while FBG levels were significantly higher, and the insulin sensitivity index was consistently lower in the model rats at 4, 8, and 12 weeks, compared with the control group. (b) Calcein double-labeling was measured using a fluorescence microscope. Arrows represent bone formation in the interval between the two injections of calcein. (c) Mineral apposition rate, mineralizing surface/BS, and bone-formation rate were analyzed using the Osteo-Measure histomorphometry system. ^∗^*P* < 0.05 vs. control, ^#^*P* < 0.05 vs. T2DM.

In this study, we established a rat model of T2DM combined with osteoporosis using intralipids and a small dose of streptozotocin, as a widely used model mimicking T2DM. Diabetes is mainly characterized by weight loss, high blood sugar, and insulin resistance (ISI), and we therefore confirmed the validity of the model by examining body weight, blood sugar level, and ISI in the model animals. Body weight was significantly lower in the model compared with normal rats, while FBG was higher and ISI was lower in the model compared with normal rats (Figure 6(a)). These results validated our animal model of T2DM.

Dynamic bone histomorphometry parameters were calculated using calcein double-labeling to evaluate the effect of melatonin on bone formation in T2DM rats. Calcein was injected twice (12 and 2 days) before the rats were sacrificed. The interlabel distance was significantly reduced in the T2DM compared with the control group (Figure 6(b)). MAR, mineralizing surface/BS, and BFR were all significantly increased in the melatonin-treated groups (50 or 10 mg/kg) compared with the T2DM group (Figures 6(b) and 6(c)).

**Figure 7:**
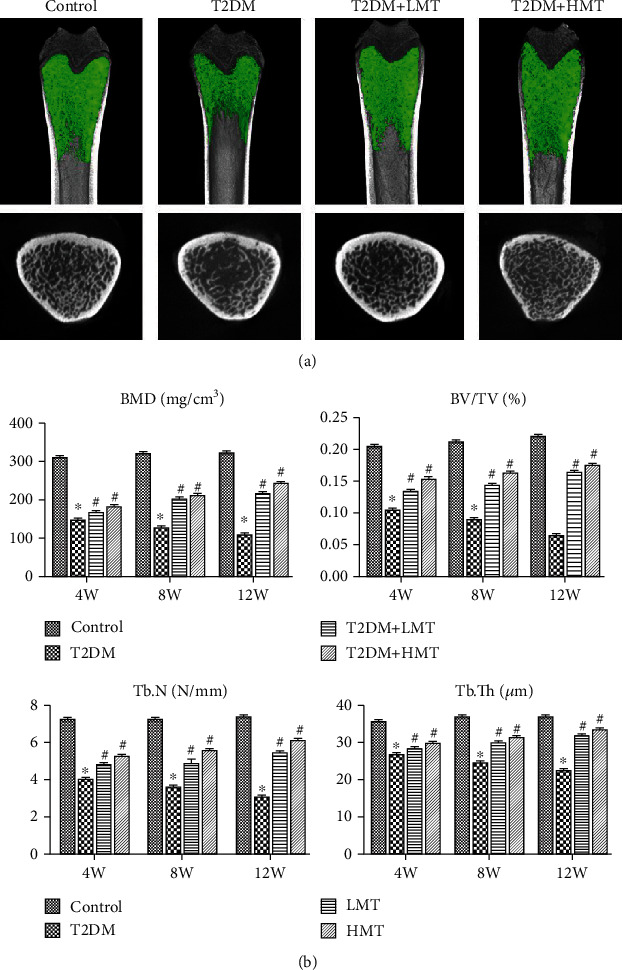
Melatonin displays protective effects on trabecular bone mass in type 2 diabetic osteoporosis. (a) Microcomputed tomography (CT) analysis of the distal metaphyseal femur region. (b) Micro-CT-based quantification within the distal metaphyseal femur region. The 3D indices in the defined region of interest were analyzed. BMD: bone mineral density; Tb.N: trabecular number; Tb.Th: trabecular thickness; BV/TV: relative bone volume over total volume. ^∗^*P* < 0.05 vs. control, ^#^*P* < 0.05 vs. T2DM.

To analyze the effects of melatonin on bone microstructure, we assessed dynamic trabecular bone formation markers, including BFR/BV and MAR, and static indexes including BMD, Tb.N, and Tb.Th. Dynamic and static analyses of the tibia revealed that bone structure was significantly worse in T2DM compared with the normal rats. We injected additional diabetic rats with high-dose melatonin (HMT, 50 mg/kg) or low-dose melatonin (LMT, 10 mg/kg) and measured the above parameters in these rats and in T2DM control rats (T2DM group). HMT and LMT both promoted the formation of trabecular bone and increased BMD, BV/TV, Tb.N, and Tb.Th (Figures 7(a) and 7(b)). These results suggested that melatonin improved the bone microstructure in rats with diabetes mellitus.

**Figure 8:**
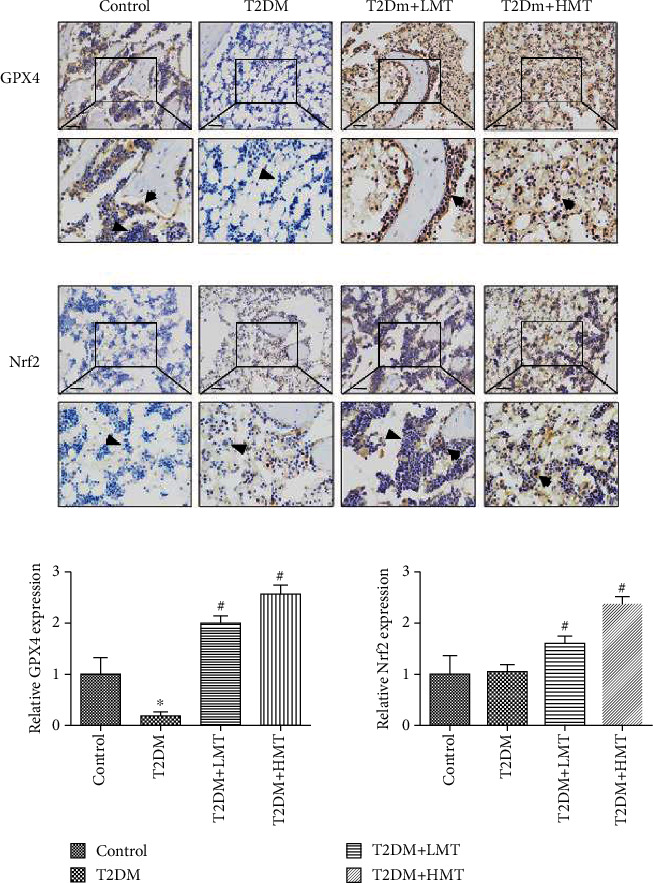
Melatonin suppresses ferroptosis via activation of the Nrf2 signaling pathway *in vivo*. GPx4 and Nrf2 expression were detected by immunohistochemistry at 12 weeks. GPx4 protein expression was significantly lower in the type 2 diabetes mellitus (T2DM) compared with the control group, and GPx4 and Nrf2 expression levels were higher in the high-dose and low-dose melatonin groups compared with the T2DM group. ^∗^*P* < 0.05 vs. control, ^#^*P* < 0.05 vs. T2DM.

GPx4 is a biomarker of ferroptosis. We detected GPx4 protein levels in bone tissue by IHC. GPx4 was widely distributed in cortical and cancellous bone. The bone ferroptosis level was significantly higher in the T2DM group compared with the control group but was lower in the HMT and LMT groups compared with the T2DM group (Figure 8). Nrf2 levels in bone tissue were also detected by IHC and were significantly higher in the HMT and LMT groups than in the T2DM group (Figure 8). These results suggested that ferroptosis was significantly increased in the bone tissues of T2DM rats, but that melatonin could reduce ferroptosis via the Nrf2/HO-1 pathway.

## 3. Discussion

There is a growing body of research on T2DOP, but the best animal model to use remains unclear [42]. The aim of the current study was to explore the mechanism of diabetes complicated by osteoporosis, which required a model that imitated the pathological process of diabetes. We therefore created a rat model of T2DOP using intralipids and low-dose streptozotocin, which has been widely used to mimic T2DM [43, 44]. We also treated osteoblasts with HG (25.5 mM) to create a diabetic microenvironment *in vitro*, as reported previously [40]. The results showed that HG could inhibit the activity of osteoblasts and bone microstructure both *in vivo* and *in vitro*.

Its complex mechanism means that the pathogenesis of T2DM has been poorly studied. We previously found that iron overload and oxidative stress were important factors in the pathogenesis of T2DOP [45, 46]. The recently identified form of programmed cell death known as ferroptosis, characterized by the accumulation of intracellular iron and lipid ROS, has aroused great interest in scientists since it was first reported in 2012 [9]. However, the study of ferroptosis is still in its infancy, and there is currently no easy or accurate way to detect the presence of ferroptosis [47]. In this study, we found that the inhibitory effect of HG on osteoblast survival could be reversed by the ferroptosis inhibitor Fer-1, and by the addition of DFO. Electron microscopy revealed that HG affected the morphology of mitochondria in osteoblasts by increasing the density of the double-layered mitochondrial membrane and decreasing inner mitochondrial cristae. This morphological change was consistent with the effects of erastin, as a positive control. HG also increased intracellular ROS, promoted the accumulation of lipid oxides, and decreased the expression level of the ferroptosis-related protein GPx4 in cells. Overall, these results indicated that ferroptosis was induced by HG in osteoblasts.

Nrf2 is a well-known transcription factor that plays a key role in antioxidation and is also considered to be an important regulatory factor for ferroptosis [48, 49]. The Nrf2/HO-1 signaling pathway was recently shown to act as an endogenous antioxidant by antagonizing oxidative stress damage in multiple organs and pathological injury mechanisms [50]. Experiments confirmed that activation of the Nrf2/HO-1 signaling pathway enhanced the expression of a variety of antioxidants and protected myocardial cells from damage caused by oxygen free radicals [51]. Sun et al. found that activation of the Nrf2 pathway protected against ferroptosis in hepatocellular carcinoma cells [32]. Activation of the Nrf2-ARE pathway thus contributed to the resistance of cancer cells to GPx4 inhibition, and inhibition of this pathway reversed the resistance of cancer cells to ferroptosis [33]. Nrf2 may protect against iron-induced injury, while long-term iron exposure resulted in iron accumulation, cytosolic ROS formation, and increased HO-1 mRNA expression, accompanied by nuclear translocation of Nrf2 and induction of its target protein NQO1 [52]. This evidence confirmed the hypothesis that Nrf2 regulates intracellular iron metabolism via the Nrf2/HO-1 axis pathway to regulate ferroptosis [48]. Consistent with previous reports, the current study demonstrated that HG induced ferroptosis in osteoblasts by inhibiting the Nrf2/HO-1 signaling pathway and promoting intracellular iron overload and lipid oxide accumulation in T2DOP.

Melatonin is a neuroendocrine hormone, mainly secreted by the pineal body, which has a variety of physiological effects including regulation of the biological rhythm, improving sleep quality, increasing immunity, and exerting antioxidative stress, antitumor, and antiaging effects [21, 53]. Numerous studies have investigated the relationship between melatonin and the NRF pathway. Deng et al. demonstrated that melatonin reduced neurological damage by activating the Keap1-Nrf2-ARE signaling pathway and reversing manganese-induced oxidative injury [41]. Moreover, melatonin activated cortical astrocytes via the Nrf2/HO-1 signaling pathway in a mouse model of cerebral hemorrhage, to increase resistance to hemin toxicity and oxidative stress [54]. In the current study, melatonin reduced intracellular lipid ROS levels and increased GPx4 activity via activation of the Nrf2/HO-1 signaling pathway, inhibiting the effects of ferroptosis induced by HG, and thus increasing the osteogenic capability of osteoblasts and bone microstructure *in vivo* and *in vitro*. *NRF2* knockdown using siRNA inhibited the beneficial effect of melatonin on osteoblast activity, further indicating that the Nrf2 signaling pathway played a key role in the intervention of osteoporosis by melatonin.

Melatonin is currently believed to improve bone density and reduce the incidence of osteoporosis [34, 55, 56]; however, the concentration of melatonin used to treat osteoporosis remains controversial. Based on the existing literature, we used five melatonin concentrations with positive and negative effects *in vivo* and *in vitro*: 10 and 50 mg/kg melatonin for *in vivo* tests and 1, 10, and 100 *μ*M melatonin for *in vitro* tests [34, 57, 58]. The results demonstrated for the first time that melatonin suppressed osteoporosis in diabetes mellitus. Unlike previous reports, the effects of melatonin were not dose-dependent, and both high and low doses improved the bone microstructure and promoted bone formation *in vivo* and *in vitro*. These results indicated that melatonin may be suitable for the treatment of T2DOP.

## 4. Conclusions

In summary, the current findings provided the first evidence for the pathway underlying T2DM. Although the relationship between ferroptosis and the Nrf2/HO-1 signaling pathway has been reported in a variety of diseases, this study provided the first results to demonstrate that HG induced ferroptosis via increased ROS/lipid peroxidation/GSH depletion in T2DOP. Importantly, melatonin significantly reduced ferroptosis and improved the osteogenic capacity of osteoblasts via the Nrf2/HO-1 pathway both *in vivo* and *in vitro*.

## Figures and Tables

**Table 1:** Primer sequences used in real-time PCR experiments.

Gene	Primer sequence 5′-3′	Product size (bp)
*NRF2*	F: GAGACGGCCATGACTGATTT	198
	R: CAGTGAGGGGATCGATGAGT	
*HMOX1*	F: GAAGAAGATTGCGCAGAAGG	181
	R: GAAGGCGGTCTTAGCCTCTT	
*ALPL*	F: GCCCTCTCCAAGACATATA	159
	R: CCATGATCACGTCGATATCC	
*OPG*	F: AGGGCATACTTCCTGTTGCC	121
	R: TGTTCATTGTGGTCCTCGGG	
*OCN*	F: CCCTGCTTGTGACGAGCTAT	90
	R: GGGCAGCACAGGTCCTAAAT	
*ACTB*	F: GTGCTATGTTGCTCTAGACTTCG	174
	R: ATGCCACAGGATTCCATACC	

bp: base pair.

## Data Availability

The data used to support the findings of this study are available from the corresponding author upon request.
